# Development and Qualification of a VSV-N IgG ELISA to Assess Vector-Directed Humoral Immunity in VSV-Vectored Vaccine Studies

**DOI:** 10.3390/vaccines14070592

**Published:** 2026-07-03

**Authors:** Morolayo Ayorinde, Claire Streatfield, Vanaja Kakarla, Faith Sigei, Rachel Bromell, Arianna Marini, Marija Zaric

**Affiliations:** 1 IAVI Human Immunology Laboratory, Imperial College London, London SW10 9NH, UK; 2 IAVI, New York, NY 10004, USA

**Keywords:** vesicular stomatitis virus, VSV, viral vector vaccines, ELISA, nucleoprotein, anti-vector immunity, assay qualification, humoral immunity

## Abstract

**Background/Objectives:** Vesicular Stomatitis Virus (VSV)-vectored vaccines represent a versatile platform for the development of vaccines against infectious diseases. Replication-competent recombinant VSV vectors in which the native glycoprotein (G) is replaced with a heterologous antigen (rVSVΔG) are widely used and have demonstrated clinical success. In addition to antigen-specific responses, these vaccines induce humoral immunity directed against vector components, which primarily reflects vector exposure and contributes to the overall characterization of vaccine-induced immunity. Standardized assays for quantifying such vector-directed responses are therefore of increasing importance. **Methods:** We developed and qualified a quantitative enzyme-linked immunosorbent assay (ELISA) for the detection of human IgG antibodies against VSV-Nucleoprotein (N). Assay development included optimization of antigen coating, blocking conditions, and detection reagents. A 10-point standard curve was established using pooled human serum, and assay performance was evaluated by assessing dynamic range, sensitivity, cut point, dilutional linearity, precision, robustness, and sample stability. **Results:** The optimized assay utilized a coating concentration of 2 μg/mL VSV-N antigen (100 ng/well) and 1% casein as the blocking buffer. The assay demonstrated a dynamic range of 0.33–41.66 arbitrary units per milliliter (AU/mL) with excellent curve fit (R^2^ > 0.996). The cut point was established at an OD_450_ of 0.286. Precision across intra-assay, inter-assay, and inter-operator evaluations met predefined acceptance criteria (≤25% CV). The assay was robust across different plate washers and readers and maintained performance following up to three freeze–thaw cycles. **Conclusions:** This study describes a robust and reproducible ELISA for quantifying anti-VSV-N IgG responses. The assay provides a fit-for-purpose tool for assessing vector-directed humoral immunity and supports standardized immunogenicity evaluations across VSV-vectored vaccine studies.

## 1. Introduction

Viral vectored vaccines have become an important platform for the development of vaccines against infectious diseases, particularly in the context of emerging pathogens [1,2]. Among these, Vesicular Stomatitis Virus (VSV), an enveloped, negative-sense RNA virus of the *Rhabdoviridae* family, has been widely utilized as a vaccine vector due to its strong immunogenicity, cytoplasmic replication, and ability to induce both humoral and cellular immune responses [3,4,5]. A commonly used approach involves replication-competent recombinant VSV vectors in which the native VSV Glycoprotein (G) is replaced with a heterologous viral antigen (rVSVΔG), enabling antigen presentation while retaining the ability to replicate. This platform has been successfully translated into clinical use, exemplified by the licensure of rVSVΔG-EBOV-GP (Ervebo^®^), an rVSVΔG-based Ebola virus vaccine [6,7,8], and continues to play a significant role in vaccine development [9,10,11,12].

In addition to immune responses directed against the encoded vaccine antigen, VSV-vectored vaccines induce immune responses against vector components. In the context of rVSVΔG systems, where the VSV G is replaced, these responses are not expected to mediate classical neutralizing anti-vector immunity. Instead, antibodies directed against internal structural proteins primarily reflect vector exposure and contribute to the overall characterization of vaccine-induced immunity. Recent clinical studies have demonstrated that antibody responses to VSV-N are robust and durable following rVSVΔG vaccination, correlate with antigen-specific responses, and can distinguish vaccinated from unvaccinated individuals, supporting their utility as biomarkers of vector exposure [5,13].

As VSV-based vaccine platforms expand, standardized assays are needed to measure vector-directed immune responses and enable consistent interpretation of immunogenicity data across studies and vaccine candidates. The VSV-Nucleoprotein (N) is a conserved structural component of the virus and represents a suitable target for assessing vector-directed antibody responses independently of the inserted antigen [14,15,16]. Measurement of anti-VSV-N IgG responses provides a means to evaluate exposure to the vector backbone and to support immunological assessments across VSV-vectored vaccine studies. As VSV-vectored vaccines progress through clinical development for multiple pathogens, including Lassa, Sudan, Bundibugyo and Marburg viruses, understanding vector-directed immune responses is becoming increasingly important. Such responses may influence interpretation of immunogenicity data, facilitate identification of prior vector exposure, and support longitudinal monitoring of vaccinated populations. Standardized approaches for measuring anti-vector immunity are therefore needed to enable comparison of results across studies and vaccine constructs.

Although human infection with VSV is rare and typically associated with mild or subclinical disease [17,18,19], the presence of anti-VSV-N antibodies may also reflect prior natural exposure, further supporting the relevance of this assay for identifying vector or virus exposure.

Here, we describe the development and qualification of quantitative ELISA for the detection of IgG antibodies against VSV-N. The assay was optimized and evaluated for key performance characteristics, including sensitivity, dynamic range, precision, robustness, and sample stability. This assay is intended as a fit-for-purpose tool to support the measurement of vector-directed humoral immunity and to facilitate harmonized immunogenicity assessments across VSV-vectored vaccine studies, including those presented within this Special Issue.

## 2. Materials and Methods

### 2.1. Study Samples

Human serum samples used for assay development and qualification were obtained from commercially available sources (Cambridge Biosciences, Cambridge, UK) and from participants enrolled in a Phase 1 clinical study evaluating a replication-competent recombinant VSV-vectored vaccine expressing Lassa virus glycoprotein (rVSVΔG-LASV-GPC) [10]. All samples were used under appropriate ethical approvals and material transfer agreements.

A pooled human serum sample with detectable anti-VSV-N IgG, generated from multiple vaccinated individuals, was used as a reference standard for calibration curve generation and as a positive control. Equal volumes of serum from four vaccinated individuals were pooled. Commercially available human AB serum (Sigma-Aldrich, Gillingham, UK, Cat No. H3667) was used as a negative control. All samples were stored at −80 °C prior to use and handled in accordance with standard laboratory procedures. Where indicated, samples were subjected to repeated freeze–thaw cycles to assess stability.

### 2.2. Reagents and Materials

Recombinant VSV-N (GenScript, Rijswijk, The Netherlands, Lot No. U658EFK200-4/P3GA001) was used as the capture antigen. The protein was expressed in *Escherichia coli* and purified by nickel affinity chromatography. The recombinant protein included an N-terminal His-tag and had a total length of 428 amino acids. Purity of the protein preparation was ≥90% as assessed by SDS-PAGE, and identity was confirmed by LC-MS. The protein concentration was 1.41 mg/mL (Bradford assay, ThermoFisher Scientific, Loughborough, UK, Cat. No. 23236), with endotoxin levels ≤ 3.9 EU/mg. The antigen was supplied in PBS containing 10% glycerol and 500 mM NaCl (pH 7.4) and stored at −80 °C. Repeated freeze–thaw cycles were avoided.

Goat anti-human Fc IgG conjugated to horseradish peroxidase (HRP) (Sigma-Aldrich, Gillingham, UK, Cat. No. A0170) was used as the detection antibody.

Phosphate-buffered saline (PBS; Sigma-Aldrich Gillingham, UK, Cat. No. D8537) and PBS containing 0.05% Tween-20 (PBS-T; prepared using Tween-20, Sigma-Aldrich, Gillingham, UK, Cat. No. P2287) were used as assay and wash buffers. Plate blocking and sample dilutions were performed using PBS supplemented with 1% casein (Bio-Rad, Hercules, CA, USA Cat. No. 1610783). Tetramethylbenzidine (TMB) substrate (SeraCare, Milford, MA, USA, Cat. No. 5120-0074) and stop solution (SeraCare, Milford, MA, USA, Cat. No. 5150-0021) were used for signal development.

High-binding 96-well microplates (Corning, Nottingham, UK, Cat. No. 3590) were used for all assays. Plate washing was performed either manually or using an automated plate washer (SkanStacker 300, Lier, Norway), and optical density (OD) measurements were acquired using a microplate reader (BioTek 800TS, Santa Clara, CA, USA).

### 2.3. ELISA Procedure

Unless otherwise specified, the following conditions were used for all ELISA procedures; variations to specific parameters were introduced during assay optimization as described in the Results.

#### 2.3.1. Plate Coating

ELISA plates were coated with recombinant VSV-N diluted in PBS to a final concentration of 2.0 μg/mL (50 μL per well; equivalent to 100 ng per well). Plates were sealed and incubated at 2–8 °C for 16–20 h.

#### 2.3.2. Plate Blocking

Following coating, plates were washed four times with PBS-T (300 μL per well) and blocked with 200 μL of blocking buffer (PBS supplemented with 1% casein) for 1 h at room-temperature (20–25 °C).

#### 2.3.3. Preparation of Standards, Controls, and Samples

A pooled human serum reference standard generated from four vaccinated individuals was arbitrarily assigned a nominal concentration of 100 AU/mL, as no international reference standard for anti-VSV-N IgG currently exists. A 10-point, two-fold serial dilution of this reference standard was prepared to generate the assay calibration curve, with each dilution assigned its corresponding nominal AU/mL value.

The assay positive control was prepared from the same pooled reference standard by dilution to a nominal concentration of 10 AU/mL (1:1000 dilution) and was included in every assay run to monitor assay performance. The negative control consisted of commercially available human serum diluted 1:100. Test samples were diluted in blocking buffer, typically starting at 1:100, with additional dilutions performed as required to ensure measured concentrations fell within the assay quantification range. Standards were prepared and plated in duplicate, while controls and samples were prepared and tested in triplicate. Plate layout is outlined in Supplementary Figure S1.

#### 2.3.4. Sample Incubation

After blocking, plates were washed twice with PBS-T. Diluted standards, controls, and samples (50 μL per well) were added to the plate and incubated for 2 h at room temperature (20–25 °C).

#### 2.3.5. Detection Antibody Incubation

Plates were washed four times with PBS-T, followed by addition of 50 μL per well of goat anti-human Fc IgG-HRP conjugate diluted 1:10,000 in assay buffer. Plates were incubated for 1 h at room temperature (20–25 °C).

#### 2.3.6. Signal Development and Detection

Following washing (four times with PBS-T), 50 μL of TMB substrate was added to each well and incubated for 10 min at room temperature (20–25 °C). in the dark. The reaction was stopped by adding 50 μL of stop solution to each well.

Optical density was measured at 450 nm with a reference wavelength of 620–650 nm within 5 min of stopping the reaction.

### 2.4. Assay Acceptance Criteria

Assay acceptance criteria were established based on a combination of empirical evaluation during assay development and qualification and standard practices for ligand-binding assays. Key performance parameters assessed included blank and control performance, precision, curve fit, and dynamic range. Acceptance criteria were defined as follows: blank optical density (OD_450_) < 0.10; negative control OD below the assay cut point (0.286); positive control recovery within 80–120% of its nominal concentration (10 AU/mL; acceptable range 8–12 AU/mL); replicate precision ≤ 25% coefficient of variation (%CV) for positive control and positive test samples analyzed in triplicate; and standard curve goodness-of-fit (R^2^) > 0.996 (Supplementary Table S1).

The dynamic signal range, defined as the difference between the upper and lower asymptotes of the four-parameter logistic (4-PL) curve (D–A), was also evaluated. A minimum acceptable D–A value of 2.37 was established as an assay acceptance criterion based on standard curve performance during assay qualification (Supplementary Table S4). These criteria were applied to all analytical runs to ensure consistent assay performance (Supplementary Table S1).

### 2.5. Data Analysis

Data analysis was performed using four-parameter logistic (4-PL) curve fitting in plate reader software (Tecan Magellan, version 7 and BioTek Gen5, version 3.08) and GraphPad Prism, version 9.

Sample concentrations were interpolated from the standard curve and expressed in AU/mL. For each sample, replicate measurements were averaged, and precision was assessed as the coefficient of variation (%CV). Samples with replicate values exceeding predefined variability thresholds (%CV > 25%) were repeated.

Samples were tested at multiple dilutions where required, and the reported concentration was derived from the dilution falling within the linear range of the assay. Samples with concentrations exceeding the upper limit of quantification were reanalyzed at higher dilutions.

Samples with OD values below the assay cut point at the lowest tested dilution (1:100) were considered seronegative and assigned a nominal value of 1 AU/mL for analysis.

All data were reported to three decimal places and reviewed against assay acceptance criteria prior to reporting.

## 3. Results

### 3.1. Assay Optimization

Assay development focused on optimization of antigen coating concentration, blocking conditions, and incubation parameters. ELISA plates were coated overnight at 2–8 °C with recombinant VSV-N at concentrations of 1 μg/mL or 2 μg/mL, followed by blocking with 1% casein, 10% fetal bovine serum, or 5% milk for 60 or 90 min at room temperature.

**Figure 1:**
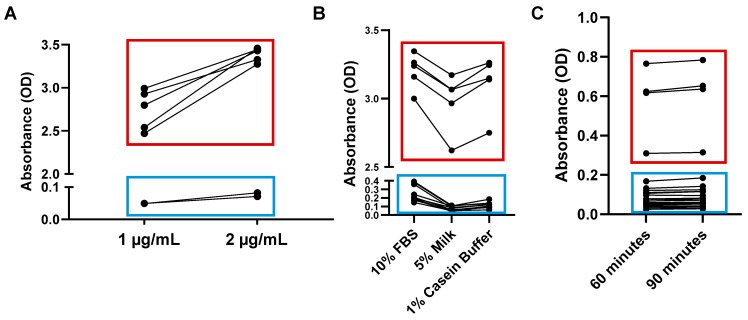
Assay optimization. (**A**) Optimization of antigen coating concentration using 1% casein as the blocking buffer. ELISA signal obtained using VSV-Nucleoprotein (N) coating concentrations of 1 μg/mL and 2 μg/mL was compared using positive (framed in red) and negative (framed in blue) human serum samples. Each dot represents an individual sample measurement (mean of triplicate wells), and lines connect the same sample tested under different conditions. (**B**) Evaluation of blocking conditions and incubation time. Blocking buffers (1% casein, 10% fetal bovine serum, and 5% milk) and (**C**) blocking durations (60 and 90 min) were assessed. Each dot represents an individual sample measurement (mean of triplicate wells), and lines connect the same sample tested under different conditions.

Evaluation using panels of positive and negative human serum samples demonstrated that coating at 2 μg/mL resulted in higher signal intensity and improved discrimination compared to 1 μg/mL (Figure 1A). Among blocking conditions, PBS with 1% casein provided the most optimal background reduction and signal-to-noise ratio (Figure 1B). Blocking durations of 60 and 90 min yielded comparable results; therefore, 60 min was selected for routine use (Figure 1C).

### 3.2. Standard Curve Performance and Quantification

A 10-point standard curve was generated using pooled human serum containing anti-VSV-N IgG, initially diluted 1:100 and serially diluted two-fold. Optical density values were measured at 450 nm and fitted using a four-parameter logistic (4-PL) regression model.

**Figure 2:**
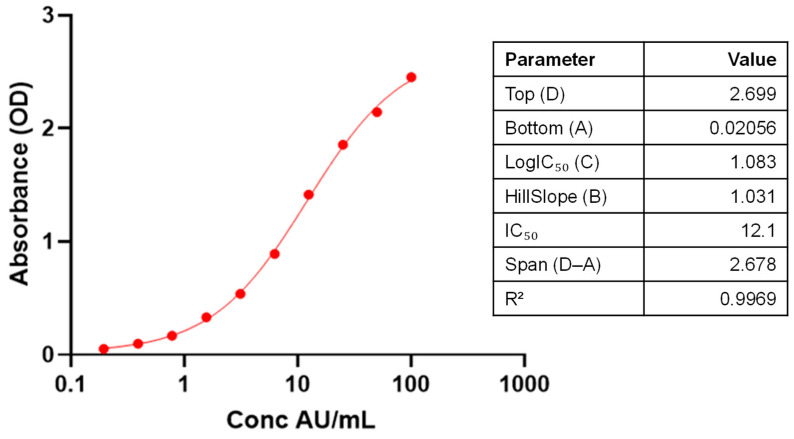
Standard curve performance. Representative 10-point standard curve generated using two-fold serial dilutions of pooled human serum containing anti-VSV-N IgG. Optical density values were fitted using a four-parameter logistic (4-PL) model. Curve fit parameters included the lower asymptote (A), upper asymptote (D), slope (B), and inflection point (C), with goodness-of-fit (R^2^) consistently exceeding 0.996. The dynamic signal range (D–A) for this representative curve was 2.678.

The assay demonstrated excellent curve fitting, with goodness-of-fit values consistently exceeding R^2^ > 0.996 (Figure 2). The dynamic signal range, defined as the difference between the upper and lower asymptotes of the standard curve (D–A), was evaluated as a measure of assay performance. Analysis of data from 17 assay development runs demonstrated that D–A values were approximately normally distributed without evidence of skewness or outliers. Based on the observed distribution, the arithmetic mean minus two standard deviations was selected empirically as the minimum acceptable D–A threshold, as this represented a conservative lower performance limit while maintaining sensitivity to identify analytical runs with suboptimal standard curve performance. Using this approach, the minimum acceptable D–A value was established as 2.37 (mean D–A = 2.79; SD = 0.21) (Supplementary Table S4). The representative standard curve shown in Figure 2 exhibited a D–A value of 2.678, exceeding this threshold. The D–A parameter reflects the usable signal window of the assay and is indicative of its ability to reliably distinguish between low and high antibody concentrations.

### 3.3. Dynamic Range, Sensitivity, and Cut Point Determination

**Figure 3:**
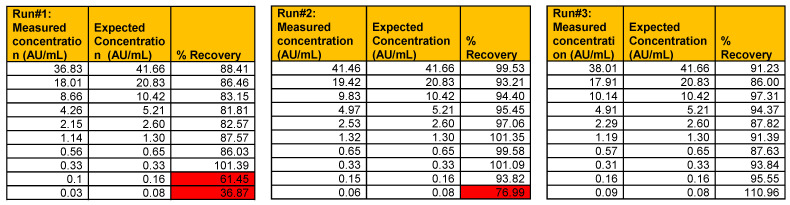
Dynamic range determination. Assessment of assay dynamic range using serial dilutions of a reference sample across independent runs. Red fields indicate values outside acceptance criteria (recovery outside 80–120%) and were excluded from the defined dynamic range. The lower and upper limits of quantification (LLOQ and ULOQ) were defined as the lowest and highest concentrations within the acceptable range and were determined to be 0.33 AU/mL and 41.66 AU/mL, respectively.

The assay dynamic range was established using serial dilutions of the pooled human serum reference standard described in Section 2.1, which was also used for calibration curve generation and as the assay positive control, across multiple independent runs. Expected concentrations were assigned based on the nominal AU values attributed to the serial dilutions of the pooled reference standard. The lower and upper limits of quantification (LLOQ and ULOQ) were defined as the lowest and highest concentrations, respectively, at which the assay demonstrated acceptable accuracy, with recovery within 80–120% of expected values and consistent performance across runs. Based on these criteria, the LLOQ and ULOQ were determined to be 0.33 AU/mL and 41.66 AU/mL (Figure 3).

**Figure 4:**
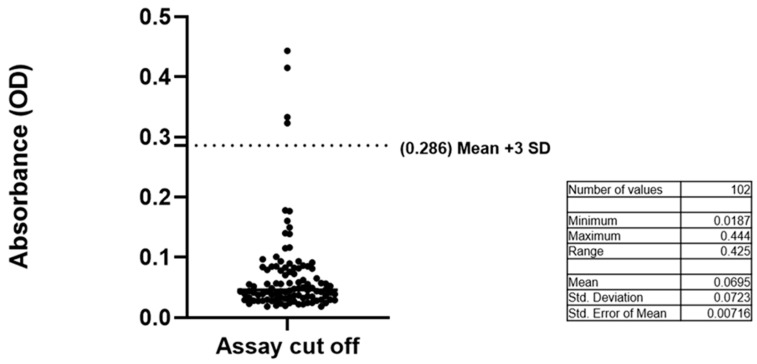
Cut point determination. Distribution of optical density (OD_450_) values obtained from 102 human serum samples not known to be exposed to VSV or rVSVΔG-based vectors. Each dot represents an individual sample measurement. The assay cut point was defined as the mean OD plus three standard deviations (mean +3 SD), indicated by the dotted line at 0.286. Summary statistics for the dataset are shown in the inset table.

The assay cut point was determined using a panel of 102 commercially obtained human serum samples from individuals with no known exposure to VSV or rVSVΔG-based vectors. Samples were evaluated across six independent assay runs. The assay cut point was established as the mean OD450 value of the negative serum panel plus three standard deviations, resulting in a cut point of 0.286 OD, which defines the threshold for distinguishing seronegative from seropositive samples (Figure 4). The mean +3 SD approach was selected to provide a conservative cut point that minimizes false-positive classification while maintaining appropriate analytical specificity for this fit-for-purpose immunogenicity assay.

### 3.4. Dilutional Linearity

**Figure 5:**
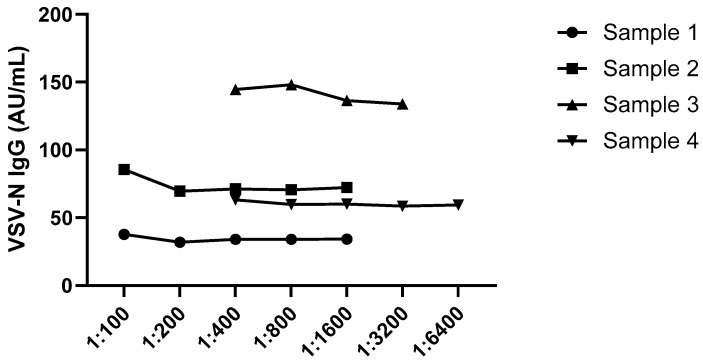
Dilutional linearity. Assessment of dilutional linearity using serial dilutions (1:100 to 1:6400) of seropositive samples. Symbols represent measured interpolated antibody concentrations at each dilution (mean of triplicate wells), and lines connect values derived from the same sample across dilutions. Dilutions producing values outside the assay range were excluded from the dilutional linearity assessment and therefore are not presented in this figure.

Dilutional linearity was assessed using two-fold serial dilutions (1:100 to 1:6400) of four seropositive samples selected to represent antibody responses spanning the assay range. Concentrations were interpolated from the standard curve and corrected for the dilution factor. Therefore, consistent AU/mL values across serial dilutions were interpreted as evidence of acceptable dilutional linearity, demonstrating that the assay can accurately quantify antibody concentrations across a wide range of responses following appropriate sample dilution (Figure 5).

### 3.5. Precision

**Figure 6:**
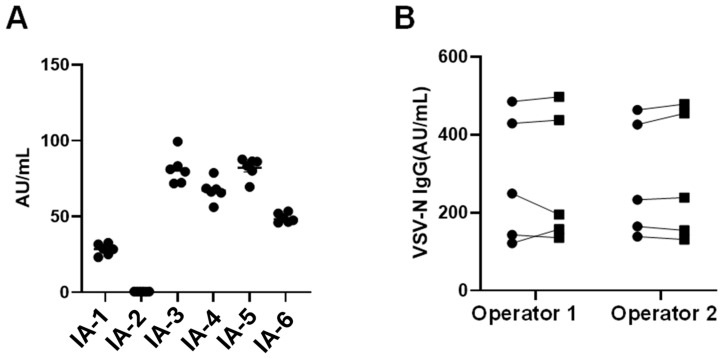
Assay precision. (**A**) Intra-assay precision assessed using replicate measurements of one negative and five positive samples (IA-1 to IA-6). Data from two independent runs are shown. (**B**) Inter-assay and inter-operator precision evaluated across independent runs performed by two operators. Symbols represent measured antibody concentrations (mean of triplicate wells), and lines connect measurements from the same sample generated by the same operator.

Intra-assay precision was assessed using six samples comprising one seronegative and five seropositive samples (IA-1 to IA-6). Each sample was tested in three independent repeat measurements within each of two assay runs, with each measurement performed in triplicate wells. Observed intra-assay precision ranged from 6.2% to 13.0% CV across the six samples evaluated (Figure 6A; Supplementary Table S2), substantially below the predefined acceptance criterion of ≤25% CV.

Inter-assay and inter-operator precision were assessed using five seropositive samples (IO-1 to IO-5) tested across two independent assay runs performed by two operators (Figure 6B; Supplementary Table S3). All inter-assay and inter-operator precision estimates remained below the predefined acceptance criterion of ≤25% CV.

### 3.6. Assay Robustness

Assay robustness was evaluated by comparing manual versus automated plate washing methods and by assessing performance across different microplate readers. For each comparison, robustness was assessed using five samples tested across four independent runs performed by two operators (two runs per operator).

**Figure 7:**
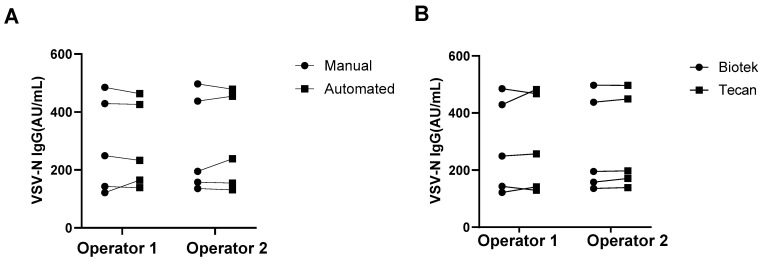
Assay robustness. (**A**) Comparison of manual versus automated plate washing methods. (**B**) Comparison of assay performance across different microplate readers. Symbols represent measured antibody concentrations (mean of triplicate wells), and lines connect measurements from the same sample tested under different conditions by the same operator.

No significant differences in measured antibody concentrations were observed between washing methods or instrumentation (Figure 7A,B), demonstrating that assay performance is robust to variations in these operational conditions.

### 3.7. Sample Stability to Freeze–Thaw Cycles

**Figure 8:**
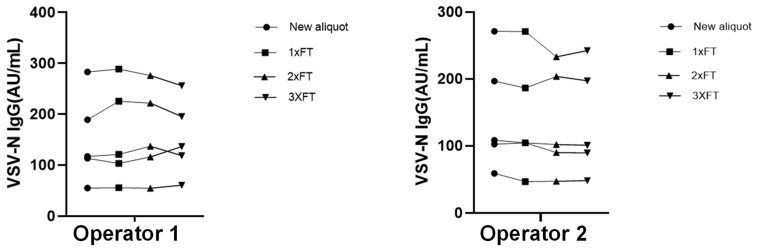
Sample stability following repeated freeze–thaw cycles. Five seropositive samples were subjected to 0 (new aliquot), 1, 2, or 3 freeze–thaw cycles before testing. For each sample, antibody concentrations were measured following each freeze–thaw condition, and the resulting concentrations are shown as individual data points (mean of triplicate wells). Lines connect measurements from the same sample across freeze–thaw conditions. Results generated independently by two operators are shown.

Sample stability was assessed by subjecting five seropositive samples to 0, 1, 2, or 3 freeze–thaw cycles prior to analysis in two independent runs performed by two operators. Measured antibody concentrations remained consistent across all freeze–thaw conditions, with variability within acceptable limits (%CV ≤ 25%), indicating that samples are stable under typical handling and storage conditions (Figure 8).

## 4. Discussion

In this study, we describe the development and qualification of a quantitative ELISA for the detection of human IgG antibodies directed against VSV-N. The assay demonstrated robust performance across key parameters, including sensitivity, dynamic range, precision, dilutional linearity, and operational robustness, supporting its use as a fit-for-purpose tool for measuring vector-directed humoral responses in VSV-vectored vaccine studies. Acceptance criteria were established based on observed assay performance during development and qualification, ensuring that thresholds were appropriate for routine application.

A recent clinical study has demonstrated that antibody responses to VSV-N are robustly induced following rVSVΔG-ZEBOV-GP (Ervebo^®^) vaccination, are maintained over time, and correlate with antigen-specific immune responses, including target-pathogen glycoprotein-directed antibodies [14], although only transient N-specific IgG was detected in another smaller study using a different assay [5]. In addition, responses to VSV-N have been shown to reliably distinguish vaccinated from unvaccinated individuals, supporting their utility as biomarkers of vector exposure [14].

The ELISA described here provides a qualified quantitative method with a defined dynamic range and sensitivity suitable for detecting and monitoring anti-VSV-N IgG responses in clinical samples. In outbreak-prone settings, measurement of antigen-specific antibodies (i.e., antibodies directed against the target pathogen glycoprotein) may not reliably distinguish between vaccination and natural infection. Detection of VSV-specific immune responses may therefore provide a complementary biomarker of vector exposure, enabling more accurate interpretation of vaccination status and its relationship to subsequent infection or disease.

Measurement of anti-VSV-N IgG thus provides a valuable tool for characterizing vector-directed immunity alongside antigen-specific responses, without interference by the heterologous antigen expressed by the vector and may also provide an indication of prior exposure to native VSV.

The assay described here demonstrated a broad dynamic range and reliable quantification of anti-VSV-N IgG. The use of a pooled human serum standard enabled consistent generation of a 10-point calibration curve with excellent goodness-of-fit. Precision assessments indicated acceptable variability across intra-assay, inter-assay, and inter-operator conditions, and the assay was shown to be robust to variations in plate washing methods and instrumentation. In addition, sample stability following multiple freeze–thaw cycles supports the applicability of this assay for use with clinical samples subjected to routine handling and storage conditions in the laboratory setting.

A recent study has employed a highly sensitive platform, such as single-molecule array (Simoa)-based assay, to characterize vector-directed immune responses following rVSVΔG-ZEBOV-GP vaccination [14]. These approaches offer markedly increased analytical sensitivity compared to conventional immunoassays, enabling detection of low-abundance antibodies. However, such technology typically requires specialized instrumentation, centralized laboratory infrastructure, and higher operational costs, which may limit their applicability in field settings. In contrast, ELISA-based methods provide a robust, scalable, and widely accessible alternative that can be readily implemented across diverse laboratory environments. In this context, the assay described here offers a practical balance between analytical performance and deployability, supporting its use in large-scale VSV-vectored vaccine studies and in settings where access to advanced platforms may be limited.

Importantly, the assay was developed as a fit-for-purpose immunogenicity assay intended to support measurement of vector-directed antibody responses in clinical vaccine studies and was not designed as a diagnostic assay. Accordingly, the qualification strategy focused on demonstrating suitability for the intended application rather than establishing diagnostic performance characteristics.

Several limitations should be acknowledged. A limitation of this assay is the use of an in-house reference standard. However, this approach is consistent with early-phase assay development and provides a reproducible and internally consistent framework for relative quantification. Future efforts may focus on further standardization, including the development of external reference materials and harmonization across laboratories to improve comparability and interpretation of results. All serum samples used in assay development and qualification were obtained from healthy adult donors; however, information regarding rheumatoid factor status, autoimmune disease history, or the presence of other potentially interfering antibodies was not available. In addition, cross-reactivity with sera from individuals exposed to related rhabdoviruses or other potentially cross-reactive infections was not evaluated. Future studies incorporating such sample panels may provide additional information regarding assay specificity. However, these evaluations were beyond the scope of the current fit-for-purpose assay qualification. Robustness assessments were limited to evaluation of operator variability, instrument variability, and sample stability under the intended assay operating conditions. The ≤25% CV acceptance criterion was selected prior to qualification based on anticipated assay variability and the intended use of the assay as a fit-for-purpose immunogenicity assay. As additional operational experience is gained across ongoing clinical studies, these performance criteria may be further refined if warranted.

This assay is intended to support immunogenicity assessments in VSV-vectored vaccine studies and can facilitate characterization of vector-directed humoral responses across different clinical settings. More broadly, standardized measurement of anti-vector immunity will be important for enabling comparisons across vaccine constructs, study populations, and clinical datasets. As additional VSV-vectored vaccines enter clinical development, standardized assessment of vector-directed immunity may become increasingly important for understanding the impact of repeated vector exposure, evaluating booster vaccination strategies, and facilitating comparison of immune responses across vaccine platforms. The availability of qualified and accessible assays will support generation of comparable datasets and may contribute to future efforts aimed at harmonization of immunological assessments across studies. The assay is currently being applied in ongoing clinical studies of VSV-vectored vaccine candidates, including rVSVΔG-LASV-GPC, rVSVΔG-SUDV-GP, and rVSVΔG-MARV-GP (targeting Lassa virus, Sudan virus, and Marburg virus respectively), supporting its applicability across multiple vaccine constructs. Given the increasing number of VSV-vectored vaccine candidates currently in clinical development, including those targeting Lassa and Marburg viruses (ClinicalTrials.gov identifiers: NCT04794218, NCT05868733, NCT07425821), standardized measurement of vector-directed immune responses will be critical for interpreting vaccine-induced immunity and addressing potential confounding effects of prior vaccination across studies. In this context, the assay described here provides a practical tool to support consistent evaluation of vector-directed immune responses and contributes to harmonized analyses across VSV-based vaccine studies.

## 5. Conclusions

A quantitative ELISA for the measurement of anti-VSV-N IgG was successfully developed and qualified for use in clinical vaccine studies. The assay demonstrated acceptable sensitivity, dynamic range, dilutional linearity, precision, robustness, and sample stability, supporting its use as a fit-for-purpose tool for assessment of vector-directed humoral immunity. This assay may facilitate standardized evaluation of anti-vector immune responses across VSV-vectored vaccine platforms and clinical development programs.

## Data Availability

The data supporting the findings of this study are available from the corresponding author upon reasonable request.

## Supplementary Materials

The following supporting information can be downloaded at: https://www.mdpi.com/article/10.3390/vaccines14070592/s1, Figure S1: Plate layout; Table S1: Assay acceptance criteria; Table S2: Intra-assay precision assessment; Table S3: Inter-assay and inter-operator precision assessment; Table S4: Derivation of the minimum acceptable dynamic signal range (D–A) acceptance criterion.
