# Supplementary material for: Development and Qualification of a VSV-N IgG ELISA to Assess Vector-Directed Humoral Immunity in VSV-Vectored Vaccine Studies
Source: Vaccines (Basel). 2026 Jul 3;14(7):592. doi: 10.3390/vaccines14070592 (PMC13416878; doi:10.3390/vaccines14070592)
Supplement: Supplementary file 1 [file vaccines-14-00592-s001.zip › vaccines-4357795-supplementary.pdf]

Supplementary Information

Supplementary Figure S1: Plate Layout

	1	2	3	4	5	6	7	8	9	10	11	12
A	Sample 1	Sample 2	Sample 3	Sample 4	Sample 5	Sample 6	Sample 7	Sample 8	Sample 9	Sample 10	Sample 11	Sample 12
B												
C												
D	Sample 13	Sample 14	Sample 15	Sample 16	Sample 17	Sample 18	Sample 19	Sample 20	Sample 21	Sample 22	Neg Control (Sigma)	Positive Control
E												
F												
G	Standard 1	Standard 2	Standard 3	Standard 4	Standard 5	Standard 6	Standard 7	Standard 8	Standard 9	Standard 10	Blank	
H												

Supplementary Table S1: Assay acceptance criteria

PARAMETER	ACCEPTANCE VALUE for IgG ELISA	COMMENT
Blank Control (OD)	< 0.10	N/A
Positive Control (Nominal concentration: 10 AU/mL)	8AU/mL<= PC <=12AU/mL	80–120% recovery of nominal concentration
Negative Control (OD)	< 0.286	N/A
%CV	≤ 25	Applies to positive control and positive test samples analysed in triplicate.<br>If %CV exceeds 25%, up to four individual data points may be excluded, subject to the following conditions:<br>• Maximum of one data point may be excluded from any triplicate.<br>• Maximum of four excluded data points per plate.<br>• No data points may be excluded from standard curve samples, the negative control or blank wells.
ULOQ	41.66 AU/mL	
LLOQ	0.33 AU/mL	
ASSAY CUT OFF (OD)	0.286	N/A
R^2	>0.996	N/A
D-A	≥2.37	N/A

Supplementary Table S2: Intra-assay Precision Assessment

Sample*	Mean (AU/mL)	SD	CV (%)
IA-1	28.30	3.69	13.02
IA-2	0.55	0.06	10.14
IA-3	81.25	10.07	12.40
IA-4	67.28	7.24	10.76
IA-5	82.21	6.77	8.24
IA-6	49.06	3.06	6.23

**A panel of representative serum samples (IA-1 to IA-6) used for the intra-assay precision assessment.*

Supplementary Table S3: Inter-Assay and Inter-Operator Precision Assessment

Sample*	Mean (AU/mL)	SD	CV (%)
IO-1	141.11	3.07	2.18
IO-2	143.01	30.36	21.23
IO-3	427.76	2.06	0.48
IO-4	474.57	15.21	3.21
IO-5	241.39	11.32	4.69

**A panel of representative serum samples (IO-1 to IO-5) used for the inter-assay/inter-operator precision assessment.*

***Supplementary Table S4:* Derivation of the minimum acceptable dynamic signal range (D–A) acceptance criterion**

Metric	Value
Number of assay runs	17
Mean D–A	2.79
SD	0.21
Minimum acceptable D–A value (mean − 2SD)	2.37
